# Synergistic Antitumor Effects of Endostar in Combination with Oxaliplatin via Inhibition of HIF and CXCR4 in the Colorectal Cell Line SW1116

**DOI:** 10.1371/journal.pone.0047161

**Published:** 2012-10-10

**Authors:** Fengyan Jin, Huifan Ji, Chunshu Jia, Ulf Brockmeier, Dirk M. Hermann, Eric Metzen, Yingqiao Zhu, Baorong Chi

**Affiliations:** 1 The First Hospital of Jilin University, Changchun, China; 2 Jilin Province Tumor Hospital, Changchun, China; 3 Department of Neurology, University Hospital Essen, Essen, Germany; 4 Department of Physiology, University of Duisburg-Essen, Essen, Germany; Univ of Bradford, United Kingdom

## Abstract

Combination treatment with endostar, a novel modified endostatin, and cytotoxic chemotherapies showed a survival benefit in Chinese clinical trials. However, the exact mechanism for this synergism remains unclear. In this study, we report for the first time that the chemokine receptor CXCR4 and the hypoxia-inducible transcription factors (HIF)-1α and HIF-2α are involved in these synergistic antitumor effects in human colorectal cancer SW1116 cells *in vitro* when endostar treatment is combined with the cytotoxic drug oxaliplatin. Under normoxia, we demonstrate that endostar and oxaliplatin treatments synergize to inhibit SW1116 cell proliferation, Matrigel adhesion and invasion by reduction of CXCR4 expression. Consistently, these antitumor abilities of endostar and oxaliplatin were markedly reduced by silencing of CXCR4 in SW1116 cells. Under low oxygen conditions (hypoxia, 1% oxygen), enhanced proliferation of SW1116 cells exposed to oxaliplatin was observed due to the emergence of drug resistance. Strikingly, endostar overcame oxaliplatin-resistance, most likely as a consequence of reduced HIF-2α and CXCR4 levels. CXCR4, is only dependent on HIF-2α, which promotes more aggressive phenotype and more significant for oxaliplatin resistance in SW1116 cells. Our data not only provide clues to aid understanding of the mechanism of the synergism of endostar and chemotherapy under either normoxia or hypoxia, but also suggests a new strategy of combination endostar and chemotherapy treatments which might potentiate therapeutic efficacies and/or counteract chemotherapy resistance.

## Introduction

Even though 5-year-survival rates of localized colorectal cancer (CRC) approach 90%, 50% of patients have developed distant metastasis at the time of diagnosis [1]. Despite new chemotherapeutic regimens and target therapies, CRC remains one of the three leading causes of cancer-related death in the worldwide [2], [3]. Metastasis and drug resistance are major problems in CRC chemotherapy. Therefore, looking for the predictors for recurrence and effective therapy counteracting drug resistance is a particular challenge for CRC. Recent studies indicate that expression levels of the chemokine receptor CXCR4 may not only predict early relapse, but also influence occurrence of drug resistance [4]–[8].

CXCR4, a seven-transmembrane G-protein-coupled receptor, acts through its specific ligand, CXCL12, leading to intracellular signaling cascades. The CXCL12/CXCR4 axis plays the critical role in HIV infection [9], B-cell development [10], stem cell mobilization and homing [11] and angiogenesis [12], [13].

There is growing evidence for CXCR4 involvement in the process of tumor progression and metastasis. CXCR4 overexpression has been identified as a negative prognostic marker in a various type of cancers, such as breast cancer, colorectal cancer and lung cancer [5], [14], [15]. Several lines of evidence support the clinical relevance of this chemokine receptor by demonstrating that CXCR4 promotes angiogenesis and site specific cancer metastasis to the favorable organs, where its ligand CXCL12 is abundantly expressed [14], [15]. Recently, a novel role of CXCR4 has emerged that reveals CXCR4 mediates resistance to endocrine therapy in human breast cancer [8] and chemotherapy in CRC [6]. Therefore, targeting CXCR4 not may only control tumor spread, but also may reverse drug resistance in cancer chemotherapy or endocrine therapy.

Drug resistance is a major problem and limitation of anticancer chemotherapy. Research into the mechanism of chemotherapy resistance has revealed that hypoxia and its transcriptional factors (HIFs) contribute to chemotherapy failure by leading to the induction of survival pathways and suppression of apoptotic potential in solid tumor cells [16]–[19]. Hypoxia mediated pathways involved in chemotherapy resistance may predict clinical response. Thus, interference with HIF function holds great promise to improve drug resistance.

Recombinant human endostatin (rhEndostatin), a 20-kDa collagen XVIII fragment is a potential angiogenesis inhibitor that shows potent anti-endothelial angiogenesis and/or anti-tumor activities *in vitro* and *in vivo* animal models [20]–[24]. However, clinical studies of endostatin were terminated at phase II in the U.S.A due to no therapeutic benefit for the progression of cancer [25]. Surprisingly, endostar, an modified endostatin bearing a 6His zinc-binding peptide at its N-terminus, shows more potent clinical effectiveness than rhEndostatin, and has been approved by the State Food and Drug Administration of China (SFDA) as a cancer drug. Endostar, exerts synergistic activities in both lung cancer and CRC when combined with chemotherapeutic agents in clinical trials [26], [27]. However, the underlying mechanism is still a mystery.

It has been reported that endostatin exerts anti-angiogenesis and anti-tumor effects in a HIF-1α dependent manner [28]. However, little is understood regarding whether endostar can reverse hypoxia-induced chemotherapy resistance. We therefore not only sought to explore the mechanisms responsible for the synergistic efficiency of endostar and chemotherapy under normoxic condition, but also to determine whether or how endostar reverses chemotherapy resistance in hypoxia.

## Materials and Methods

### Cell Culture

The colorectal cancer cell line SW1116 (ATCC CCL-233) were grown and subcultivated in Leibovitz’s L-15 medium (Gibco). HEK 293T cells were cultured in Dulbecco-modified Eagle medium (DMEM, Gibco). All culture media were supplemented with 10% fetal calf serum, penicillin (100 units/ml) and streptomycin (100 µg/ml). Cells were grown in a humidified incubator at 37°C and 5% CO_2_. Hypoxic culture conditions were achieved in a hypoxic chamber (Toepffer Lab Systems, Göppingen Germany) by inflation of N_2_ and CO_2_ into the air-filled chamber until 1% O_2_ was reached.

### Chemokines, Antibodies, Reagents and Plasmids

Recombinant human endostar was generously provided by Shandong Simcere Medgenn Bio-Pharmaceutical Company, China. Oxaliplatin (OXA) was obtained from ShenZhen Haiwang Pharmaceutical Company, China. Recombinant human/rhesus macaque/feline CXCL12/SDF-1α and monoclonal mouse anti-CXCR4 (clones 12G5 and 44716) antibodies were purchased from R&D Systems. Polyclonal rabbit anti-CXCR4 antibody was obtained from Abcam (Cambridge, UK). Monoclonal mouse anti-α-tubulin antibody was from Santa Cruz Biotechnology. The hydroxylase inhibitor, Dimethyloxalylglycine (DMOG) was purchased from Alexis Biochemicals. Plasmids pLKO.1-shRNA-CXCR4-1 (Mission® TRC shRNA TRCN0000256863), pLKO.1-shRNA-CXCR4-2 (Mission® TRC shRNA TRCN0000256866), pLKO.1-shRNA-HIF-1α-1 (Mission® TRC shRNA TRCN0000003810) and pLKO.1-shRNA-HIF-2α-2 (Mission® TRC shRNA TRCN0000003806) were from Sigma.

### Transfection and Lentiviral Transduction

Transient transfections were conducted using GeneJuice® transfection reagent (Merck KGaA, *Darmstadt*, Germany) in a ratio 3∶1 (µl reagent/µg DNA), as recommended by the manufacturer. For production of recombinant lentivirus, 1×10^6^ HEK 293T cells were co-transfected with 6 µg of target vector pLKO.1-shRNA, 4 µg of psPAX2 (12260; Addgene) and 2 µg pMD2G-VSVG (12259; Addgene) and incubated for 48 hours. The medium containing recombinant lentivirus was harvested, filtered through a 0.45 µm filter unit (Millipore; Schwalbach, Germany) and stored at –80°C. Functional virus titer was calculated by transfecting HEK 293T cells with limiting virus dilutions of the green fluorescent protein (GFP)-carrying vector pWPXL (12257; Addgene) and subsequent quantification of GFP positive cells by fluorescence microscopy. On average, the viral titer was 1×10^6^ transduction units/ml. For transduction, 2×10^5^ target cells were incubated for 20 hours with lentiviral supernatant (2×10^6^ transduction units) containing polybrene (8 µg/ml).

### CXCR4 Semiquantitative Reverse Transcription-PCR (RT-PCR) Analysis

Total RNA was isolated from SW1116 cells by Trizol Reagent (Invitrogen). 1 µg/ml total RNA from each sample was used for reverse transcription by using EasyScript Reverse Transcriptase (TransGen Biotech, China) in a total volume of 20 µl. Reverse transcription was performed for 30 min at 42°C followed by 10 minutes at 85°C. 2 µl of RT product were subjected to PCR. The following primers were used: GAPDH forward 5′-CAAGGTCATCCATGACAACTTTG-3′; GAPDH reverse 5′-GTCCACCACCCTGTTGCTGTAG-3′ (product size 489 bp), CXCR4 forward 5′-GGCCCTCAAGACCACAGTCA-3′; CXCR4 reverse 5′-TTAGCTGGAGTGAAAACTTGAAG-3′ (product size 352 bp). Amplifications were run in 25 µl volume using Tap PCR MasterMix (Bioteke Corporation, China) for 35 cycles of 30 seconds denaturation at 94°C, 30 seconds annealing at 55°C and 30 seconds elongation at 72°C. The PCR amplification was followed by a 10 minute final extension at 72°C. GAPDH was used as the housekeeping gene.

### Western Blot

Whole cell lysates from SW1116 were prepared in NP40 lysis buffer containing protease inhibitor cocktail (Roche, Mannheim, Germany). Proteins were resolved on 7.5% SDS-polyacrylamide gels and electroblotted onto PVDF membranes. After transfer, blocking of unspecific binding sites was achieved by incubation in TBST (50 mM Tris/HCl, 150 mM NaCl, 0.5% Tween 20, pH 7.2) containing 5% skimmed milk. For the incubation steps with primary and HRP-conjugated secondary antibodies, antibody concentrations were used as recommended by the manufacturer. Detection was performed with the ECL kit (GE Healthcare; Munich, Germany).

### Colony Formation Assay

200 SW1116 cells were seeded into individual wells in 24 well plates and grown overnight. Recombinant human endostar (100–400 µg/ml) and/or oxaliplatin (0.625 µg/ml) were added and cultured for 7 days. After washing in PBS for 3 times, cells were fixed using methanol and stained by Giemsa. The numbers of colonies were counted and photographs were taken.

### Methylthiazol Tetrazolium (MTT) Assay

1×10^4^ SW1116 cells were seeded into individual wells in 96 well plates and cultured overnight. Cells were treated with recombinant human endostar (100–400 µg/ml) for 3 days at 37°C, in the absence or presence of oxaliplatin (25 µg/ml, 1 day). Following that, 20 µl MTT (5 mg/ml) was added into each well and incubated for 2–4 hours. MTT solution was sucked out and cells were washed with PBS for 3 times. 150 µl DMSO/per well was added to dissolved MTT completely. OD was determined by plate reader at the wavelength of 570 nm.

### Proliferation Assay

2×10^5^ SW1116 cells were seeded into a 6 well plate. Recombinant human endostar (200–400 µg/ml), or oxiliplatin and their combinations were added to the cells and cultured in 1% O_2_ condition for 3 days. For proliferation assays after HIF-1α, HIF-2α knockdown, 1×10^5^ SW1116 cells which transduced with the appropriate vectors and cultured in 1% O_2_ condition for 3 days. The viable cells were counted.

### Matrigel Adhesion Assay

A 96 well plate was coated with Matrigel (1∶3 dilution) and 1% BSA (as control) overnight. Matrigel was piped out and 1% BSA was added into wells and incubated for 1 hour. 1×10^4^ cells were seeded onto Matrigel or BSA, and incubated for 2 hours. The non-adherent cells were thoroughly washed off using 3 washes with PBS. 20 µl MTT solution (5 mg/ml) and 100 µl culture medium were added and incubated for 2–4 hours. OD was determined by plate reader at the wavelength of 570 nm. Adhesion rate (%) = [(OD _sample_/OD _control_)−1]×%.

### Matrigel Invasive Assay

1×10^4^ cells were added into the upper compartment of 24-well transwell inserts (8 µm) which were coated with Matrigel (1∶3 dilution). 100 ng/ml SDF-1α was added into the lower chamber and incubated for 24–48 hours. Matrigel and non-migratory cells on the top side of the surface were removed. The migrated cells on the bottom side of the membrane were counted in 12 standardized fields at 20×10 magnification.

### Immunofluorescence Cytochemistry

Cells were fixed either with PBS+0.1% paraformaldehyde (PFA for cell surface staining) or 1∶1 acetone/methanol (intracellular staining), blocked by immersion in 2% bovine serum albumin (BSA) and incubated 2 hours at room temperature in anti-CXCR4 (15 µg/ml; MAB172, R&D Systems) Immunofluorescence was revealed by immersion in Alexa Fluor 488-labeled secondary antibody. Slides were analyzed by using a Zeiss LSM510 confocal microscope with 20×/0.75 or 40×/1.3 oil immersion lens (Carl Zeiss, Heidelberg, Germany).

### Statistical Analysis

All experimental procedures were repeated a minimum of three independent times. Data are expressed as mean ± S.D. values. Comparisons between two groups were evaluated with two-tailed unpaired t-tests. Comparisons between more than 2 groups were analyzed with one-way ANOVA. Statistical analysis was performed using GraphPad Prism 5. *P*<0.05 was considered statistically significant.

## Results

### Endostar, in Combination with Oxaliplatin, Synergistically Inhibits SW1116 Cells Proliferation, Matrigel Adhesion and Invasion

**Figure 1:**
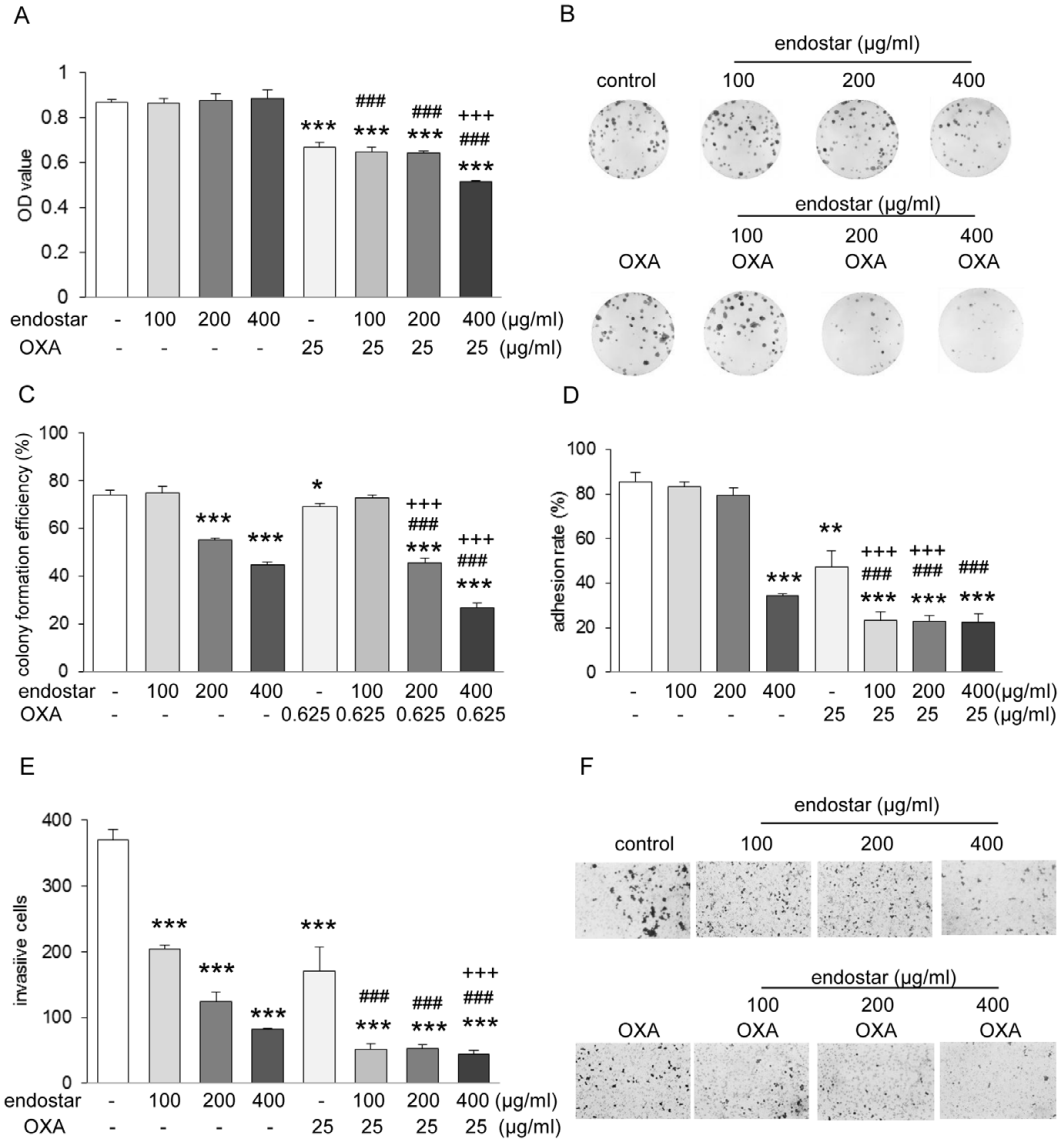
Endostar and oxaliplatin synergistically inhibited SW1116 cells proliferation, Matrigel adhesion and invasion. (**A**) Proliferation assay, (**B–C**) colony formation, (**D**) Matrigel adhesion and (**E**) Matrigel invasion of SW1116 cells exposed to endostar (100–400 µg/ml), oxaliplatin (OXA) and combination. In (**A**), cells were seeded and exposed to different concentration of endostar for 3 days, OXA for 1 day. The viable cells were determined by MTT assay. ***p<0.001 versus control; ^###^ *p*<0.001 versus their respective endostar; ^+++^ *p*<0.001 versus OXA. In (**B–C**), cells were seeded onto 24 well plate and treated with 100–400 µg/ml endostar or 0.625 µg/ml OXA or their different combinations for 7 days. Representative microphotographs are shown in (**B**). *p<0.05/**p<0.01/***p<0.001 versus control; ^###^ *p*<0.001 versus their respective endostar; ^+++^ *p*<0.001 versus OXA. In (**D**), cells pretreated with endostar (3 days), OXA (1 day) or both were trypsinized, seeded onto Matrigel or BSA coated 96 well plate, and incubated for 2 hours. Adherent cells were detected by MTT. **p<0.001/***p<0.0001 versus control; ^###^ *p*<0.001 versus their respective endostar; ^+++^ *p*<0.001 versus OXA. In (**E and F**), cells pretreated with endostar, OXA or both were seeded onto Matrigel coated transwell plate (8.0 µm) and cultured 24 hours. After removing the non-migratory cells, the migratory cells on the bottom side of membrane were quantified. ***p<0.001 versus control;^ ###^ *p*<0.001 versus their respective endostar; ^+++^ *p*<0.001 versus OXA (n = 3). Representative microphotographs are shown in (**F**).

First, we exposed SW1116 cells to a concentration range of oxaliplatin for 24 h and 72 h to obtain IC_50_ and IC_25_ values. To evaluate whether endostar in combination with chemotherapy exerts antitumor effects synergistically, we administered various concentrations of endostar alone or in combination with oxaliplatin which was kept at a constant IC_25_ and performed proliferation, Matrigel adhesion and invasion assay. As shown in Fig.1A, in the absence of oxaliplatin, endostar alone did not inhibit SW1116 cell proliferation. However, a significant inhibition of cell proliferation was found when cells were treated with 400 µg/ml endostar combined with oxaliplatin. Cell colony formation has been found to be a more sensitive parameter than cell viability [29]. In SW1116 cells treated with 200 µg/ml or 400 µg/ml of endostar, colony formation was inhibited by 22.8% or 37.9%, respectively. Combined treatment of endostar with oxaliplatin more significantly suppressed colony formation by 36.6% and 62.1% respectively (Fig.1B and 1C). These results demonstrated that endostar combined oxaliplatin have a synergistic effect on SW1116 cell proliferation.

Adhesion and invasion are the crucial steps in the process of tumor metastasis. We chose to examine adhesion on and invasion through Matrigel, a more complex matrix that serves as a more physiological substrate. Treatment with 100–200 µg/ml endostar did not show significant change in adhesion. However, 400 µg/ml endostar diminished cells adhesion to Matrigel markedly (Fig.1D). Strikingly, even treatment with 100–200 µg/ml endostar with oxaliplatin showed a dramatic decrease in adhesion to Matrigel, suggesting that the combination of endostar with oxaliplatin has a synergism on adhesion of SW1116 cells to Matrigel. We next examined the effect of endostar or oxaliplatin on the invasion of SW1116 cells through Matrigel. As shown in Fig.1E and F, endostar reduced SW1116 cell invasion through Matrigel in a dose-dependent manner at the concentration of 100–400 µg/ml. A more pronounced effect of endostar on invasion was found in cells exposed to endostar and oxaliplatin together than that in the cells treated with endostar alone.

Our data reveal that endostar not only has a direct effect, but also synergistically attenuates tumor proliferation, adhesion to and invasion through Matrigel in combination with the cytotoxic chemotherapeutic drug, oxaliplatin.

### The Chemokine Receptor CXCR4 Mediates the Antitumor Effect of Endostar and its Synergistic Effect with Oxaliplatin

**Figure 2:**
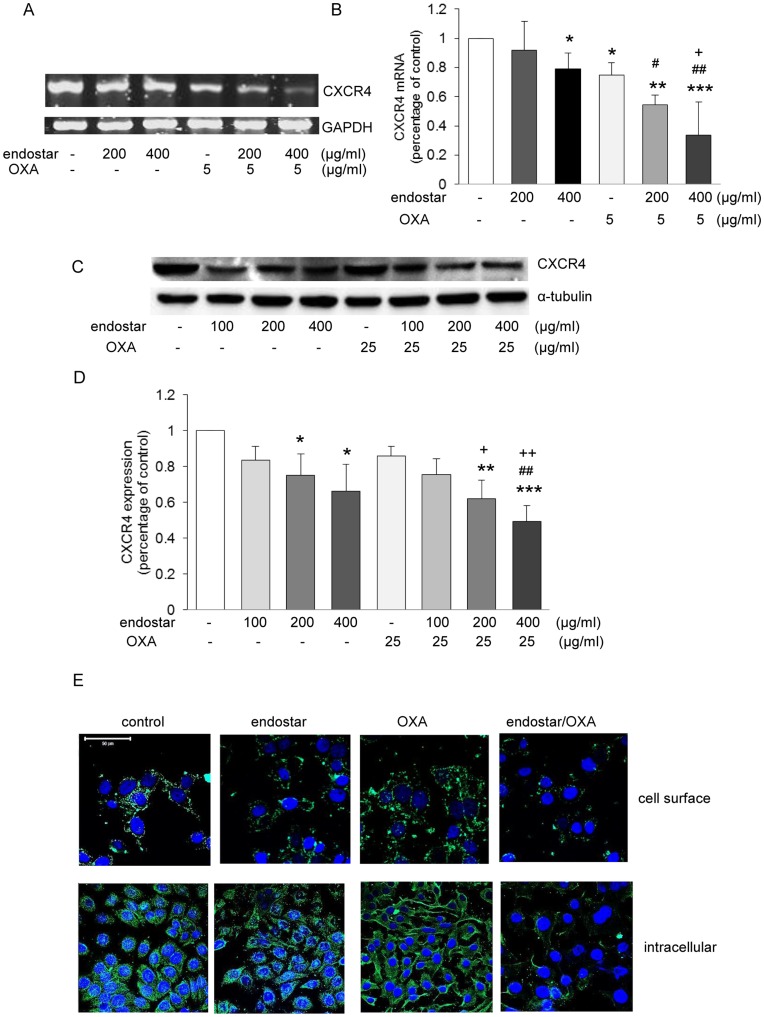
The inhibitory effect of endostar and its synergism with OXA is a consequence of reduced CXCR4 signaling. (**A–B**) CXCR4 and housekeeping gene GAPDH mRNA expression were determined by RT-PCR. *p<0.05/**p<0.01/***p<0.001 versus control; ^#^ *p*<0.05/^##^ *p*<0.01 versus their respective endostar; ^+^ *p*<0.05 versus OXA. (**C–D**) Western blots for CXCR4 and the house keeping protein α-tubulin of whole cell lysates of cells treated with endostar (100–400 µg/ml, 3 days), 25 µg/ml OXA (1 day) and their combination. *p<0.05/**p<0.01/***p<0.001 versus control; ^##^ *p*<0.01 versus their respective endostar; ^+^ *p*<0.05/^++^ *p*<0.01 versus OXA. (**E**) Confocal immunofluorescence microscopy showing cell surface and intracellular CXCR4 expression under control conditions and after endostar or OXA exposure. Scale bar, 50 µm (n≥3).

To explore the possible mechanisms mediating the antitumor effects of endostar and its synergism with oxaliplatin, we investigated whether the chemokine receptor CXCR4 is involved in the antitumor action of endostar or oxaliplatin. SW1116 cells were treated with endostar and/or oxaliplatin for 3 days and CXCR4 mRNA expression in these cells was assessed by RT-PCR. As shown in Fig.2A–B, 200–400 µg/ml endostar reduced CXCR4 mRNA markedly, and when endostar was combined with oxaliplatin, further reduction of CXCR4 mRNA was found. Oxaliplatin alone had no effect on CXCR4 protein expression. However, when SW1116 cells were treated with endostar and oxaliplatin, further reduction of CXCR4 expression was observed (Fig.2C–E). Moreover, we found endostar decreased CXCR4 expression in cell membranes and cytoplasma, indicating CXCR4 degradation may contribute to endostar-induced reduction in CXCR4 expression (Fig. 2E).

### A Pivotal Role of CXCR4 for Tumor Growth, Adhesion and Invasion in SW1116 Cells

**Figure 3:**
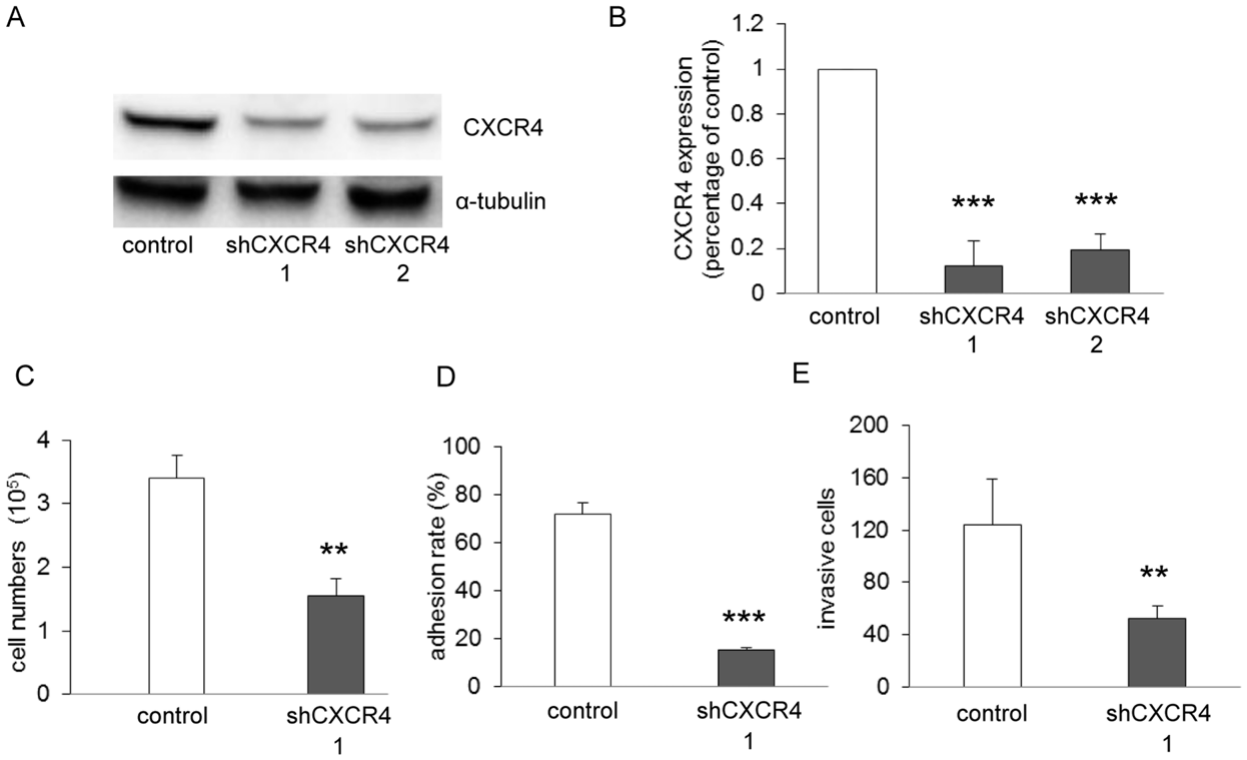
CXCR4 signaling promotes SW1116 cells proliferation, Matrigel adhesion and invasion. SW1116 cells were transduced with pLKO.1-shRNA-CXCR4. Control cells were transduced with vector pLKO.1. (**A–B**) Western blots of whole cell lysates for CXCR4 expression of SW1116 after CXCR4 knockdown. ***p<0.001 versus control. Proliferation (**C**), Matrigel adhesion (**D**) and invasion assay (**E**) were performed, showing CXCR4 contributes to proliferation, adhesion and invasion in SW1116 cells. **p<0.01/***p<0.001 versus control (n = 3).

To assess whether CXCR4 plays a direct role in tumor activities, we silenced CXCR4 in SW1116 cells by CXCR4 shRNA (Fig.3A–B) and examined the function of CXCR4 knockdown on tumor proliferation, adhesion and invasion. We found that knockdown of CXCR4 retarded cell growth (Fig.3C), diminished cell adhesion to Matrigel (Fig.3D) and invasion through Matrigel (Fig.3E) to a comparable level. These results demonstrated that CXCR4 signalling in SW1116 plays a critical role in cell growth and metastasis.

### Endostar Overcomes Hypoxia-mediated Oxaliplatin Resistance through Inhibition of the HIF-1α - or HIF-2α/CXCR4 Pathway

**Figure 4:**
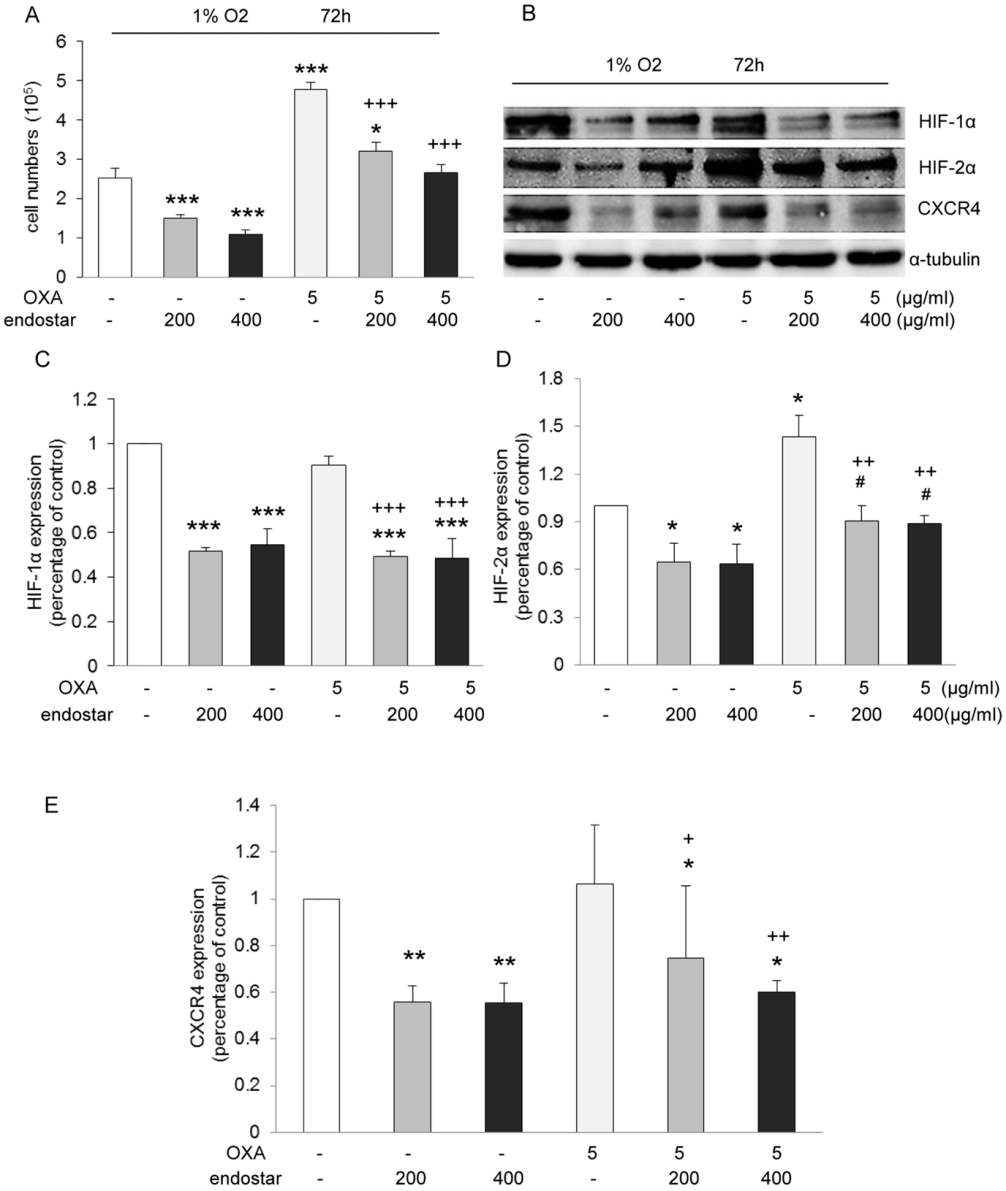
Chemotherapy resistance induced by hypoxia was reversed strongly by combining with endostar. SW1116 cells were cultured with endostar or OXA, either alone, or in combination with OXA on 1% O_2_ condition for 3 days. (**A**) Hypoxia survival assay, *p<0.05/***p<0.001 versus control; ^+++^ *p*<0.001 versus OXA (n = 3 independent experiments). Note that endostar inhibited SW1116 cell survival under hypoxia. Hypoxia induced OXA resistance under the same condition. Endostar potently reduced OXA-induced chemotherapy resistance. (**B–E**) Western blots of whole cell lysates for HIF-1α, HIF-2α, CXCR4 and housekeeping protein α-tubulin expression of SW1116 cells treated with endostar, OXA or combinations under hypoxia. *p<0.05/**p<0.01/***p<0.001/versus control;^ #^ *p*<0.05 versus their respective endostar; ^+^ *p*<0.05 versus OXA. Noted HIF-2α has more pronounced effect on inhibiting proliferation and invasion under hypoxia, in comparison with HIF-1α (n≥3).

Endostatin, has been found to attenuate angiogenesis by inhibiting HIF-1α activity [28]. However, there is evidence showing that the anti-angiogenic activity of endostatin can also be HIF-1α independent [30]. Therefore, we conducted proliferation assay of SW1116 cells exposed to endostar and/or oxaliplatin under hypoxic condition and examined whether endostar reduces CXCR4 expression via inhibition of HIF-1α and/or HIF-2α signalling. As shown in Fig.4 A, endostar treatment alone was sufficient to inhibit SW1116 growth under hypoxia. However, the same concentration of oxaliplatin as that in normoxia caused a significant increased proliferation, indicating hypoxia reduced SW1116 cells sensitivity to oxaliplatin treatment. Importantly, endostar administration strongly reversed the resistance of SW1116 cells to oxaliplatin.

We further observed decreased HIF-1α protein expression level in the hypoxic SW1116 cells with endostar treatment (Fig. 4B–D). Likewise, robust reduction of HIF-2α and CXCR4 was also found (Fig. 4B, D and E). Treatment with oxaliplatin results in a corresponding increase of HIF-2α under hypoxia (Fig. 4B and D). Interestingly, when combining endostar and oxaliplatin, HIF-1α, HIF-2α and CXCR4 protein decreased markedly (Fig. 4B–E). These data show that endostar exerts dramatic anti-proliferation effect under hypoxic condition via suppression of HIF-1α, HIF-2α and CXCR4 expression. More importantly, these data demonstrate the role of HIF-2α in mediating hypoxia-induced oxaliplatin resistance and suggest that repression of HIF-2α might contribute to overcoming oxaliplatin resistance under low oxygen conditions.

**Figure 5:**
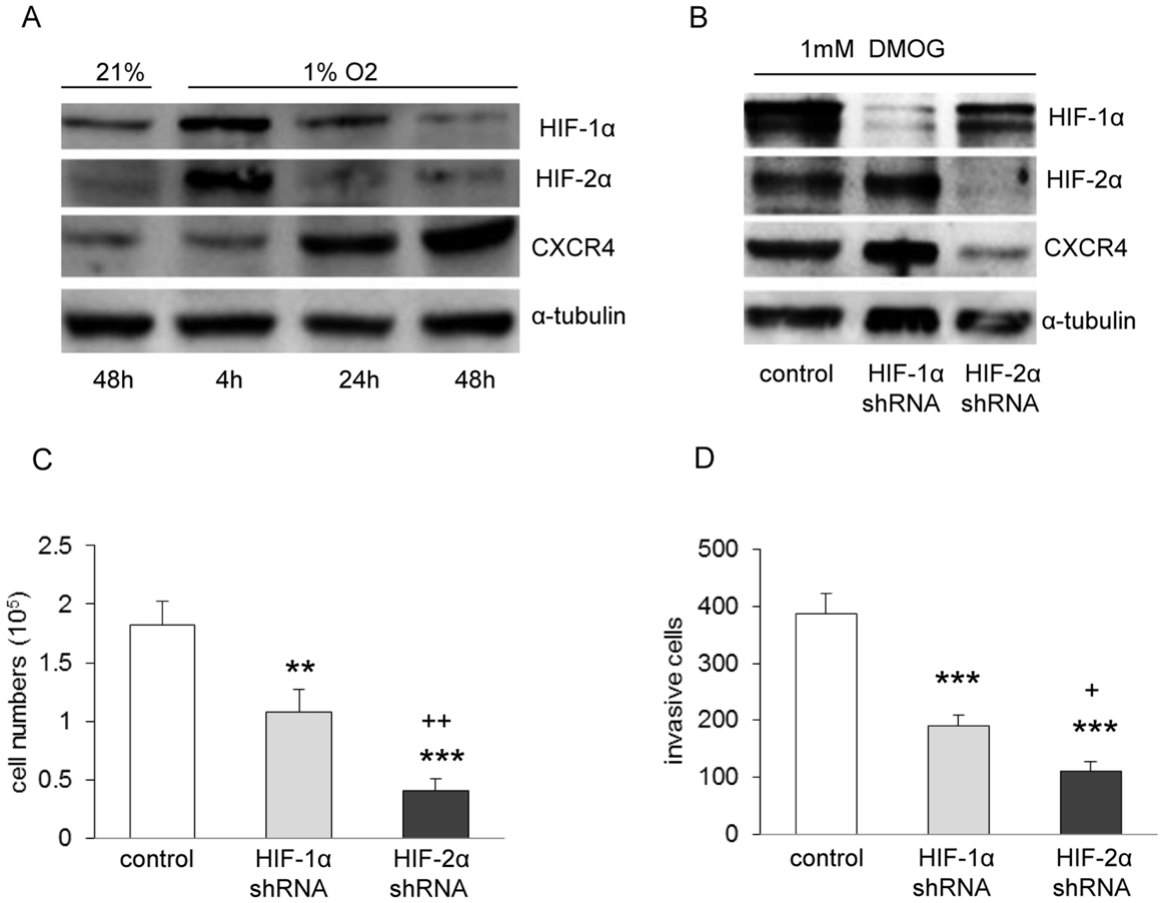
CXCR4 is hypoxia inducible, but only the target gene of HIF-2α. (**A**) Western blots of whole cell lysates for HIF-1α, HIF-2α and CXCR4 expression under hypoxic condition, demonstrating hypoxia increases CXCR4 abundance. (**B**) Western blots of SW1116 cells expressing shHIF-1α and shHIF-2α. The cells were treated with 1 mM DMOG for 4 h. Proliferation (**C**) and Matrigel invasion assay (**D**) were conducted in hypoxia. **p<0.01/***p<0.001 versus control; ^+^ *p*<0.05/^++^ *p*<0.01 versus HIF-1α control, demonstrating both HIF-1α and HIF-2α contribute to SW1116 cells survival and invasion, whereas HIF-2α inhibited proliferation and invasion to a greater extent than HIF-1α.

We have demonstrated that CXCR4 plays a key role in SW1116 cell proliferation, adhesion and invasion (Fig.3 C–E). To validate the functional significance of HIF-1α and HIF-2α, we used the same method to directly inhibit HIF-1α and HIF-2α with shRNA in hypoxic SW1116 cells (Fig.5 A), which resulted in significant reduction of proliferation (Fig.5 C) and invasion (Fig.5 D). Furthermore, shRNA against HIF-2α inhibited proliferation and invasion to a greater extent than shRNA against HIF-1α (Fig.5 C and D). Interestingly, CXCR4 is upregulated under hypoxia (Fig.5 A), but only HIF-2α knockdown reduced CXCR4 abundance (Fig.5 B), demonstrating that CXCR4 is a target gene of HIF-2α in the SW1116 cell line. Taken together, we demonstrated that endostar overcomes hypoxia-mediated oxaliplatin resistance through HIF-2α/CXCR4 pathway.

## Discussion

Endostar, a newly developed compound, shows more potent clinical effectiveness than rhEndostatin in Chinese clinical trials [26], [27]. Particularly, synergistic antitumor activities were observed when endostar was used in combination with chemotherapeutic agents [26], [27], raising the question about the mechanism underlying this synergism.

Using custom microarrays which cover over 90% of the human genome [21], it has been reported that about 12% of genes are significantly regulated in human microvascular endothelial cells by endostatin, suggesting this reagent is a broad spectrum inhibitor of angiogenesis, Previous studies have demonstrated that a number of mechanisms contribute to the anti-angiogenesis or anti-tumor activities of endostatin, including interference with integrins, E-selectin and several metalloproteinases [24], [31], [32]. By showing reduced proliferation, Matrigel adhesion and invasion synergistically following combined endostar and oxaliplitin treatment, we now provide possible mechanism for the synergistic effects of endostar and oxaliplitin. Thus, CXCR4 expression was remarkably reduced by endostar. Further reduction of CXCR4 was observed following the combination of endostar and oxaliplatin.

CXCR4, together with its ligand CXCL12, is involved in tumor angiogenesis, directional metastasis, as well as resistance to chemotherapy and endocrine treatments for cancer therapy [5], [6], [8], [14]. Supporting the role of CXCR4 in tumor activities in SW1116 cells, we herein silenced CXCR4 signaling by transducing with pLKO.1-shRNA-CXCR4 and demonstrated that CXCR4 plays a critical role in cell growth, Matrigel adhesion and invasion. Indeed, the abundance of CXCR4 was diminished on the surface of SW1116 cells following endostar and oxaliplatin treatments, providing an explanation for the reduced tumor growth and metastasis.

Hypoxia is an important feature in many solid tumors including CRC. An hypoxic microenvironment favors more aggressive cancer cell phenotypes, promotes malignant progression of cancer [33], and also induces drug resistance to chemotherapy or radiotherapy [16]–[19], [34]. The key transcriptional factors to low oxygen concentrations, HIF-1α or HIF-2α, are involved in the cell signaling bypass the effects of chemotherapy and radiotherapy [16]–[19], [34]. Thus, interference with HIF function holds great promise to improve drug resistance.

Endostatin, has been shown to exert anti-angiogenesis and anti-tumor effects in a HIF-1α dependent manner [23], [28]. In this regard, we wondered whether endostar could still have a synergistic effect with oxaliplatin on tumor growth under hypoxic condition. By demonstrating that this combination not only dramatically suppressed SW1116 cell proliferation, but also overcame hypoxia induced oxalipatin resistance, we provide further insights into the effects of endostar under hypoxic condition.

In our study, endostar results in decreased HIF-1α, HIF-2α and CXCR4 expressions which lead to diminished cell survival of SW1116 cells under hypoxic conditions. In contrast, oxaliplatin enhanced cell growth by increasing HIF-2α accumulation, indicating HIF-2α mediates a significant proportion of oxaliplatin resistance. Our results indicate that CXCR4, as a target gene of HIF-2α, may also be responsible for this resistance. To the best of our knowledge, reversal of hypoxia induced chemoresistance by endostar through HIF-2α/CXCR4 has never been shown.

Our results suggest that endostar treatment alone may be superior to combination treatment with chemotherapy if a surrogate marker is well identified for defining chemotherapy resistance. It will not only potentiate therapeutic efficacies, but also escape from toxicities of chemotherapy. Because reliable markers for patient selection are still lacking, combination of endostar and chemotherapy provides an alternative therapeutic strategy. Future studies will be necessary to determine the therapeutic utility of endostar in overcoming chemotherapy resistance by using drug-resistant cellular models under normoxic condition. It would also be of interest to identify the biomarkers to predict chemotherapy resistance as a means to select patient for single endostar therapy.
